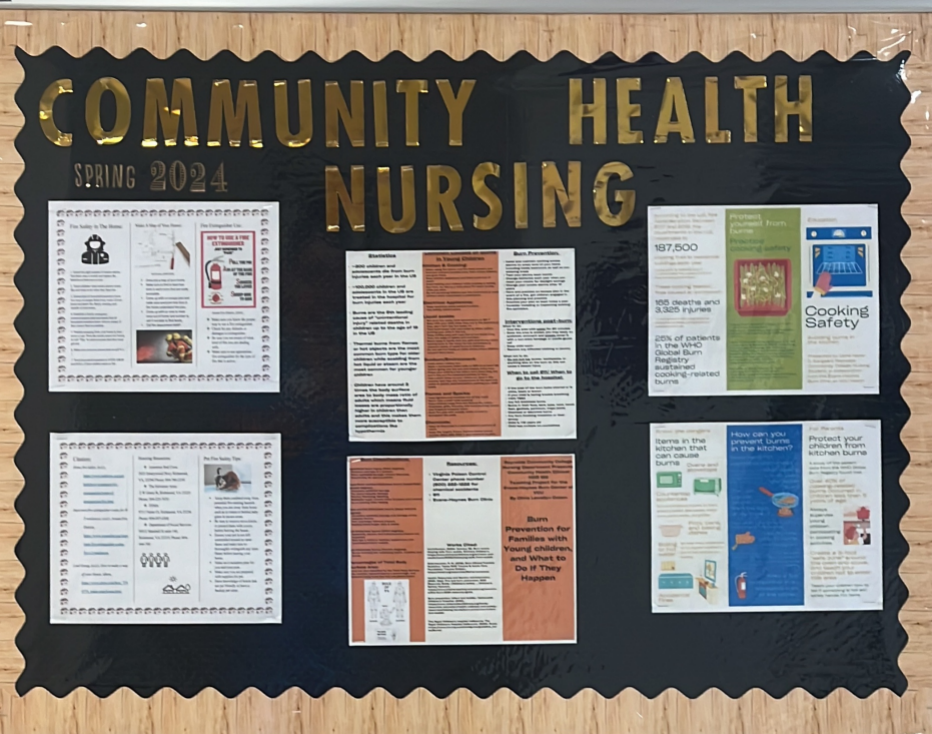# 919 Community Health Nursing in the Burn Clinic

**DOI:** 10.1093/jbcr/iraf019.450

**Published:** 2025-04-01

**Authors:** Jodi Brown

**Affiliations:** Evans-Haynes Burn Center

## Abstract

**Introduction:**

Lillian Wald, founder of the Henry Street Settlement (1893) in New York City, invented the term “Public health nursing”.

The Burn Clinic is the perfect environment to not only foster community awareness but an excellent opportunity to see patients from all ages and levels of society.

Since this was the first time the Burn clinic had Community health clinical rotation, no template for the students existed, so one was created.

**Methods:**

At the beginning of the year 2024, The Burn Clinic was asked to teach community health nursing students as a rotation. An introductory power point on Burn care basics and wound care was sent to all the incoming students. Since this was a new offering, a guide was created to mirror our existing orientation manual. The meeting was set with the professor and the four students who were coming. They received and introduction a tour of the Burn Clinic and the inpatient Unit. We collaborated with the Burn unit to get them in on every procedure possible. Working together with the Doctors, Nurses, OT (Occupational Therapy), PT (Physical Therapy), and the operating room for a broad and diverse exposure. The Case worker took the students on grand rounds and showed them the discharge criteria and how that process played out. In our clinic we see everything from the first wounds to follow-up care and long-term scar management. The pilot was a success, commended for giving the students an excellent experience and the Burn Clinic was asked to do it again this fall. We are extremely excited about this project and believe it can be done regularly as a Community Health Nursing rotation, covering all aspects from newborn to geriatric patients and from diverse economic and geographic backgrounds.

**Results:**

The students had a wonderful experience, and the school wants to make this a regular rotation for Community Health. The projects the students presented were amazing, each one focused on a different venue. They were all based on the patients they saw and cared for. The evaluations of their experiences were all unique in what they got out of the experience.

**Conclusions:**

This is a wonderful way to introduce Burn Care to future nurses, it also helps give a new perspective and insight into prevention and awareness. The projects were unique and well thought out. Covering the type of patient, they took care of and including ways to treat and prevent the types of Burns the patients they interacted with experienced. The patients also enjoyed interacting with the students and taking an interest in their care.

**Applicability of Research to Practice:**

Having community health students in the Burn Clinic is a wonderful way to show all the steps involved in Burn care. I think this can pilot many other programs in other facilities and communities that would be beneficial for students, burn care, and awareness overall.

**Funding for the Study:**

N/A